# History and Domestication of *Saccharomyces cerevisiae* in Bread Baking

**DOI:** 10.3389/fgene.2020.584718

**Published:** 2020-11-11

**Authors:** Caitlin Lahue, Anne A. Madden, Robert R. Dunn, Caiti Smukowski Heil

**Affiliations:** ^1^Department of Biological Sciences, North Carolina State University, Raleigh, NC, United States; ^2^Department of Applied Ecology, North Carolina State University, Raleigh, NC, United States; ^3^Center for Evolutionary Hologenomics, The GLOBE Institute, University of Copenhagen, Copenhagen, Denmark

**Keywords:** *Saccharomyces cerevisiae* (Baker’s yeast), bread, baking, domestication, industrial, yeast

## Abstract

The yeast *Saccharomyces cerevisiae* has been instrumental in the fermentation of foods and beverages for millennia. In addition to fermentations like wine, beer, cider, sake, and bread, *S. cerevisiae* has been isolated from environments ranging from soil and trees, to human clinical isolates. Each of these environments has unique selection pressures that *S. cerevisiae* must adapt to. Bread dough, for example, requires *S. cerevisiae* to efficiently utilize the complex sugar maltose; tolerate osmotic stress due to the semi-solid state of dough, high salt, and high sugar content of some doughs; withstand various processing conditions, including freezing and drying; and produce desirable aromas and flavors. In this review, we explore the history of bread that gave rise to modern commercial baking yeast, and the genetic and genomic changes that accompanied this. We illustrate the genetic and phenotypic variation that has been documented in baking strains and wild strains, and how this variation might be used for baking strain improvement. While we continue to improve our understanding of how baking strains have adapted to bread dough, we conclude by highlighting some of the remaining open questions in the field.

## Introduction

Bread baking in the home has experienced a surge in interest in recent years, heightened during mandatory stay at home orders during the COVID-19 pandemic (McCarron, 2020). Grocery store shelves were emptied of flours and commercial yeast (Mak and Slate Magazine, 2020), and thousands of people began sourdough starters, using spontaneous inoculation of yeast in bread and water (Aviles, 2020; Nichols, 2020). This trend in home baking comes on the heels of an increasing desire from commercial bakers to use different types of grains and to develop more interesting, complex flavors in their baked products (Glover et al., 2010; Steensels and Verstrepen, 2014). A similar phenomenon has occurred in the craft brewing industry, which has led to an explosion of research into the history of commercial brewing strains and the use of alternative yeasts (Gallone et al., 2016, 2019; Fay et al., 2019; Langdon et al., 2019). However, the yeasts used for bread baking have received considerably less attention than their beer and wine making cousins.

Leavened bread is made via two main processes. The first is the addition of commercial baker’s yeast, *Saccharomyces cerevisiae*, to dough. This yeast comes from pure cultures bought (or more rarely maintained) by bakers and bakeries. The second is the creation and maintenance of a “starter,” by allowing yeasts and bacteria to spontaneously inoculate a mix of milled grains (e.g., flour) and water. Such starters can be very old, having been passed from one human generation to the next, but many are made from scratch in the kitchen of the baker. The inoculating yeast in starters can be a mix of commercial or domesticated strains, such as *S. cerevisiae*, and/or environmental (or “wild”) strains which are undomesticated and found in non-human related environments (Johnson et al., 2004; De Vuyst and Neysens, 2005).

The first process, the use of commercial yeast, is dominated by a small handful of *S. cerevisiae* strains, such as those manufactured by Fleischmann’s, Red Star, and SAF. The second process, reliance on starters, is more complex and varied. Starters are a rich microbial community, the composition of which can vary due to human culture, geography, individual baker, type of grains used, timing of sampling in the starter’s history, and many other factors (Minervini et al., 2012; Ercolini et al., 2013; De Vuyst et al., 2014, 2016; Carbonetto et al., 2018). Different yeast species are found in different starters. However, *S. cerevisiae* are present in the majority of sampled sourdough starters. The strain diversity of *S. cerevisiae* in starters is largely unknown, particularly in breads from non-Western cultures (Pulvirenti et al., 2004; Vrancken et al., 2010; Lhomme et al., 2016).

In light of a renewed excitement from home and commercial bakers, combined with new insights from increased worldwide sampling and whole genome sequencing, we seek to highlight the genetic basis of *S. cerevisiae* adaptation to dough, both in the context of commercial yeasts and sourdough starters. Much of this review is filtered through the lens of European history and baking strains, which gave rise to several of the commercial baking strains used today. With that caveat, the emphasis of this paper is to catalog known genetic differences between bakery strains of *S. cerevisiae* compared to wild and other industrial strains. We start with a brief history of risen bread and baking yeast domestication. We focus on the particular conditions that bread yeast face, and the key genes and pathways known to be important in these conditions with a particular concentration on maltose utilization, osmotic stress tolerance, and aroma production. We conclude with a look at methods of bioprospecting and bioengineering for better baking, and key questions that the field can address moving forward.

## The Rise of Baking: A Brief History of Bread

Scientists tend to describe the discovery of the role of yeast in fermentation in relation to the visual discovery of the morphology of the yeast organisms by J. H. van den Broek in 1859 (Sicard and Legras, 2011). Yet, bakers have been using yeast for the production of food and beverages for thousands of years and, in doing so, were well aware of their reliance on living beings, which were very often named, even if their means of describing those beings was not the same as that now employed by later scientists. The earliest known records of yeast risen bread come from Ancient Egypt in 1300–1500 BCE (Samuel, 1996; Sicard and Legras, 2011) and China in 500–300 BC (Shevchenko et al., 2014). However, it is likely that organized reliance on organisms for fermentation is far older. Amato and colleagues, for example, have recently argued that early hominins likely fermented fruits using yeasts, as early as a million years ago (Dunn et al., 2020). In this context, we suspect that the reliance on yeast for grain fermentation, including that associated with bread making, is much older than current dates based on archaeological samples. If, as has been frequently argued, beer and bread making pre-date the origin of agriculture, we might imagine that so too does increased reliance on specific yeasts. However, it is noteworthy that recent genomic evidence suggests that the canonical beer and bread yeast, *S. cerevisiae*, originated in China before moving west 16–14 tya via the route which would become the Silk Road (Wang et al., 2012; Duan et al., 2018; Peter et al., 2018; Fay et al., 2019). This is just before the origin of agriculture, roughly in line with the timing of the oldest bread yet documented (which might or might not have been leavened) (Arranz-Otaegui et al., 2018). This suggests that fermentations happening outside of Asia prior to 16–14 tya likely relied on other species of yeast (not *Saccharomyces*).

Each ancient human culture that relied upon yeasts and other microbes for fermentation would have employed specific techniques for ensuring the presence of those microbes. For this review, we will focus primarily on Western bread. Evidence for the simplest method for baking leavened bread derives from an Old Kingdom tomb dating to between 2450 and 2401 BCE (Moussa et al., 1977, p. 153; Samuel, 1989). It involves mixing and kneading the dough in a bowl and baking directly on the fire’s hot ashes, and was likely used by peasants and workers in later eras (Samuel, 1989). During all of the Kingdoms, molds were also used, with the dough either being poured into the mold, or in later periods, shaped (Samuel, 1989; Baking Ancient Egyptian Bread, 2018). Other sources propose a pre-fermented mix prepared by roasting malt loaves, crushing them, mixing them with water and whole grains (likely the yeast source) and allowing it to ferment before straining it through a straw mat, although the precise time period method was used remains unclear (Frey, 1930). The dough was kneaded by walking on it, like one might press grapes, baking the loaves in large ovens lined with bricks, stone slabs, or in rare cases, iron depending on the era (Frey, 1930).

Frey (1930) hypothesizes that knowledge of these fermentation and baking methods passed from Egypt and Babylon to ancient Greece and ancient Jewish cultures (Frey, 1930). From Greece, the knowledge passed to Rome, where they kneaded dough by hand instead of by foot (Frey, 1930). Pliny the Elder records that professional bakers did not appear in Rome until 168 BCE, after a war with King Perseus (Pliny the Elder; Frey, 1930). However, reality was undoubtedly more complex, whether with regard to the spread of bread-making, the grains used in bread-making (Verberg, 2019) or the techniques for sharing and managing yeasts and starters. The intensive study of historic, pre-historic and ethnographic records relating to the care of yeasts and starters would be very rewarding. The spread of fermented beverages such as wine and beer, which were linked to bread making through the sharing of yeasts between brewers and bakers, is better studied (Frey, 1930; Sicard and Legras, 2011). Because devices and vessels were often used to store yeasts from beer to later (even months later) make bread (Verberg, 2019), it is possible that ancient yeast proteins, genomes, and maybe even living cells, may be recoverable (and would prove a rich data source for comparative analysis). However, the burden of proof to document that the yeasts that have been discovered are truly ancient is (and should be) very high.

The first “commercial” production of yeast, that is the growing of yeast for the sole purpose of selling it to others, arose in the 1700s, but unlike modern pure culture production, it was more the art of keeping a continuous colony of fermenting yeast in dough or hops for use in brewing more than baking (Frey, 1930). Prior to the introduction of these “commercial” yeasts, the primary yeast source for bakers and housewives was the yeasty foam or dreg waste collected from completed beer fermentations, and were sold directly by breweries (Frey, 1930). In the 1780s and 1790s, the development of compressed yeast began to appear in England, Germany, and Netherlands (Frey, 1930). Early recipes for commercial pressed yeasts consisted of a pressed block of fermented hops, rye, and malt, or some mixture of the three depending on the country and local producer, and could contain as little as 4–6% yeast, which would consist of multiple strains as well as any associated bacteria (Frey, 1930). By the early 1800s, these compressed yeasts were outcompeting the excess or spent yeast from brewers. In addition to compressed yeast, records from 1771 provide a simple drying method for yeast, which required mixing yeast and wood ash together before placing it in the sun to dry further (Frey, 1930). Dried yeast was first sold commercially in Vienna in 1822. Other yeast preservation methods besides drying included bottling yeast, covering the yeast with oil, and burying it several feet underground to keep it cool (Frey, 1930).

The word “yeast” was not linked to fermentation until 1859. J. H. van den Broek, working in Utrecht, Netherlands, identified vegetative cells that existed and replicated in fermenting media, which he dubbed yeast (Frey, 1930; Barnett, 2000). The word yeast derives from Late Old English *gist*, a cognate to the Middle German words *gest*, meaning dregs or dirt, and *jest*, meaning foam, as well as the Old High German word *gesan/jesan*, meaning to ferment (Barnhart, 1995). All of these cognates refer to where yeast could be found and, to some extent, the manifestations of its presence (the foam). Louis Pasteur hypothesized that yeast could be purified using tartaric acid, which he hypothesized would kill unwanted bacteria and yeast, and recommended using pure cultures for fermentation (Frey, 1930; Gélinas, 2010). The tartaric acid method, however, only removed bacteria from yeast mixtures, but did not prevent wild spoilage yeasts from replicating (Frey, 1930). Emil Christian Hansen, director of the Carlsberg Laboratory in Copenhagen, took up the work of purifying yeast strains based on the work of Pasteur and the 1870 work of Oscar Brefeld, who proposed pure cultures could be derived from single cells (Ling, 1909; Frey, 1930). While Hansen succeeded in 1879, later publishing his work in 1883, few accepted purified strains under the belief that purified strains could not remain pure, and that the taste would worsen in the absence of the products (what we would now call metabolites) of the bacteria that had been removed (Ling, 1909; Frey, 1930). It would take 20 years before pure cultures became common. The impure “yeast” used before Hansen typically contained multiple strains of yeast, as well as a variety of species of bacteria (Frey, 1930).

In the mid to late 1800s bakers began to value “pure” cultures (Frey, 1930). It was in this context that John C. Pennington patented a method, in 1879, that used a microscope to check if the yeast was a pure culture without bacterial contamination (Gélinas, 2010). The first known patent that followed Pasteur’s insistence on sterility of both media and equipment was in 1891, by Alfred Jörgensen, director of his own lab by the same name, and Axel Bergh, directory of his own lab in Stockholm, Sweden and owner of several breweries (Gélinas, 2010; Grönberg, 2019). Their patent used a sterile aeration system, thus maintaining strain purity and enhancing growth (Gélinas, 2010). Sterile media and equipment are required to maintain pure cultures, and are now standard in modern yeast production. By the early 1900s, better aeration methods and the invention of centrifuges (which replaced filters) increased production capability (Frey, 1930). This, in turn, allowed the expansion of the commercial baking industry, and by the 1920s commercial yeast as we know it was born (Frey, 1930; Gélinas, 2010).

While our main focus in this review is on Western bread, we note that Asian and African breads have a rich history; the future study of which will undoubtedly enrich our understanding of yeast history, ecology and evolution. In China, fermented sourdough bread has been an important diet item for at least the past 2000 years (Liu et al., 2018). Steamed sourdough bread is currently the most popular traditional fermented wheat product, accounting for 40% of consumed wheat in China, and is a popular breakfast item (Kim et al., 2009; Li et al., 2015; Liu et al., 2018). India has a variety of leavened and unleavened flatbreads (Mir et al., 2014). The oldest evidence of bread as of writing dates are the charred remains of flatbread dating back to 14,400 BCE from the Shubayqa1 dig site in northeastern Jordan (Arranz-Otaegui et al., 2018); whether this bread was partially leavened is, as of yet, unclear.

## Signatures of Domestication in *Saccharomyces cerevisiae* Baking Strains

Until quite recently, large-scale genomic studies of baking strains have been non-existent, and more work is still needed to tie together the history of bread with the genetic history of these strains. This gap in knowledge is beginning to be rectified, with new phylogenetic analyses of commercial and sourdough baking strains illuminating a polyphyletic origin of baking strains (Peter et al., 2018; Bigey et al., 2020). Bakery strains surveyed thus far are largely from European isolates, and fall into two major clades, suggesting at least two domestication events leading to commercial baking strains and sourdough baking strains, respectively (Bigey et al., 2020). Within these two clades, both noted for their mixed origins, the bakery strains are interspersed with strains isolated from many sources including wine, sake, clinical, and natural environments. Despite the intertwined history of beer and breadmaking, the phylogenetic relationships of beer and bread strains are largely separated. There are a few exceptions, including African maize dough strains clustering with African beer strains, and a subset of beer strains clustering with mixed origin bakery strains (Bigey et al., 2020). Surprisingly, no sequenced bakery strains cluster with Ale beer strains, although there is evidence of Ale beer strain introgression in some of the bakery strains (Bigey et al., 2020).

There are systematic differences between strains used in baking, strains associated with other fermentations, and strains not associated with human environments. This is true even though baking strains are not monophyletic, suggesting convergent adaptation to the dough fermentation environment. Baking strains tend to have more complex genetic architecture compared to laboratory strains (Steensels et al., 2014a), and fewer genes are differentially expressed in baking strains compared to domesticated wine and beer strains during dough fermentation (Aslankoohi et al., 2013). Commercial baking strains tend to be a higher ploidy (up to 68% of baking strains have a ploidy above 2n) and have higher rates of aneuploidy (up to 17% of baking strains are aneuploid) than wild and semi-wild sourdough strains (up to 35% are polyploid or aneuploid) (Bigey et al., 2020). Tetraploidization appears to have occurred at least once in the domestication of commercial baking strains, and translates to a significantly faster start of fermentation compared to diploid strains (Bigey et al., 2020). This trait was perhaps selected upon by bakers, giving rise to the numerous commercial baking strains with tetraploidy.

Polyploidy and aneuploidy are common signatures across all industrial *S. cerevisiae* (whether associated with bread, beer, or other fermentation environments). A higher ploidy leads strains to have lower sporulation efficiency, lower spore viability, and unstable mating types, especially compared to laboratory strains which are selected for easy and rapid reproduction (Liti et al., 2009; Borneman et al., 2011; Warringer et al., 2011; Steensels et al., 2014a). The side effects caused by higher ploidy complicate efforts to use traditional breeding and genetic modification techniques for strain improvement, which we discuss in more detail in the Bioengineering section, below. Thus, while higher ploidy appears to be beneficial for strain adaptation to the baking environment, it also hinders further strain improvement by limiting the types of techniques that can be used.

The ultimate signature of the evolution of bread yeasts is whether bakery strains display better dough fermentation performance (where “performance” can be defined in nearly as many ways as there are bakers) than do non-bakery strains. Common metrics to analyze dough fermentation performance include a variety of measurements related to carbon dioxide (CO_2_) production, cell growth during fermentation, and dough height, as well as metrics related to human safety, consumption, and distribution, such as lack of biogenic amine production, volatile organic compounds, bread texture, and freeze/thaw tolerance (De Vuyst and Neysens, 2005; Aslankoohi et al., 2016) and, of course, tastes, flavors and aromas (and the features associated with them).

There are general indicators of domestication shared amongst industrial strains used in bioethanol, bread, beer, and wine (Spor et al., 2009; Sicard and Legras, 2011; Randez-Gil et al., 2013). Intriguingly, many industrial strains from non-bakery origins can achieve similar CO_2_ production during dough fermentation compared to bakery strains (Aslankoohi et al., 2013). The simplest explanation for this observation is that there is a tradeoff between CO_2_ production and other bread fermentation traits. For example, attempts to increase CO_2_ production by manipulating expression of metabolic enzymes have generally failed, as increases in CO_2_ come at the cost of traits like decreased growth yield (Schaaff et al., 1989; Navas et al., 1993). This might translate to longer fermentation times and smaller loaves, and have a detrimental effect as yeast provide nutrition and flavor (Olsson and Nielsen, 2000; Bekatorou et al., 2006; Birch et al., 2013a, b).

The most convincing evidence to date of bread-specific domestication comes from a survey by Bigey et al., of a collection of sourdough strains, commercial bakery strains and non-bakery strains of diverse origins and genetic groups. They show that sourdough and commercial baking strains produce significantly more CO_2_, both in rate and in total, compared to non-bakery strains. Sourdough and commercial baking strains also had a shorter lag time than non-bakery strains, meaning they started fermenting and raising the dough earlier than did non-bakery strains (Bigey et al., 2020). This study also identified differences between sourdough strains and commercial bakery strains. Commercial strains achieved a faster fermentation onset than did sourdough strains, but sourdough ultimately achieved higher population sizes. Overall, these results support the hypothesis that commercial baking strains and at least some sourdough strains (bearing in mind that there may be a lot of strain diversity in starters) were domesticated and are better adapted to their environment than other strains.

## Important Traits and Associated Genes for Bread Baking

In this next section, we explore the particular environmental pressures that *S. cerevisiae* must handle during dough fermentation, and the desirable characteristics for optimizing *S. cerevisiae* baking strains. We address maltose utilization and glucose repression; osmotic stress; glycerol, trehalose, and proline accumulation; and aromatic compound production (Table 1).

**TABLE 1:** Key baking traits and involved genes.

Process name	Key genes	Use in baking	Strain traits	Citations
Maltose utilization	MAL loci: MALR; MALT; MALS	Needed to utilize maltose, the primary sugar in bread.	Baking strains tend to have multiple copies of MAL loci and/or MAL genes.	Duval et al., 2010; Bigey et al., 2020
Glucose suppression	MIG1	Glucose suppression shuts down maltose utilization, delays the start of fermentation, and decreases overall gasing ability.	Deletion of MIG1 has conflicting results, with glucose repression decreasing in some and increasing in others.	Keleher et al., 1992; Treitel and Carlson, 1995; Olsson and Nielsen, 2000
Osmotic stress	HOG1; AQR1; STL1; GDP1	Increased internal glycerol concentration aids cell survival; external glycerol aids in dough elasticity and thus gas retention.	HOG pathway is upregulated during dough fermentation; evidence of selection on osmosensor genes in strains used in other fermentations, but has not been examined for baking strains.	Albertyn et al., 1994; Hohmann, 2009; Aslankoohi et al., 2013, 2015; Brewster and Gustin, 2014; Heitmann et al., 2018
Trehalose and proline accumulation	TPS1; TPS2; NTH1; ATH1; MPR1; MPR2	Trehalose and proline increase cell survival and thus fermentation ability after freezing stress, for dough storage, and drying stress, during dried yeast processing.	Strains with increased trehalose and proline synthesis/retention have higher survivability.	Bell et al., 1992, 1998; Kopp et al., 1993; Alizadeh and Klionsky, 1996; Lewis et al., 1997; Samuel et al., 2000
Aromatic compounds	Varied	Yeast produced metabolites can change bread’s aromatic profile.	Individual strains can have significantly different aromatic profiles, with baking strains having had off-flavors/aromas bred out through artificial selection.	Olsson and Nielsen, 2000; Birch et al., 2013a, b; Aslankoohi et al., 2016; Dzialo et al., 2017

### Maltose Utilization and Glucose Suppression in Bread Dough

The fermentation process that results in dough rising relies on *S. cerevisiae* consuming sugar and producing ethanol and CO_2_. *S. cerevisiae* prefers easily fermentable monosaccharides such as glucose and fructose, however, many bread doughs have a very low sugar content (with the exception of high sugar doughs). As a result, the *S. cerevisiae* in dough must use more complex sugars such as maltose. Maltose is a disaccharide composed of two glucose molecules joined by an α(1→4) bond, obtained by the breakdown of starch by amylase enzymes naturally found in grains. The ability to ferment maltose is variable across species and strains of yeast (Bell et al., 2001; Houghton-Larsen and Brandt, 2006; Naumova et al., 2013), with commercial baking strains more adapted to maltose utilization than are non-bakery strains (Bell et al., 2001).

Maltose use is conferred by one or more functional MAL loci. There are five unlinked, subtelomeric MAL loci (MAL1-MAL4, MAL6) in *S. cerevisiae*. Each MAL locus includes the genes MALR, which encodes a transcriptional regulatory protein, MALT, which encodes maltose permease, and MALS, which encodes maltase (Naumov et al., 1994; Olsson and Nielsen, 2000; Houghton-Larsen and Brandt, 2006). MAL1 on chromosome VII is likely the ancestral locus from which other MAL loci are derived, as all examined *S. cerevisiae* strains and its closest relative *Saccharomyces paradoxus* have MAL1 at this position (Duval et al., 2010). While MAL loci generally exhibit high sequence and functional similarity, there are several different maltose utilization phenotypes that have been described (Duval et al., 2010). Some MAL alleles may even be non-functional, for example, the lab strain S288C has two MAL loci (MAL1 and MAL3), but cannot ferment maltose due to non-functional MALR genes (Day et al., 2002a, b). The extent of allelic variation of MAL loci and resulting phenotypes could be revisited in light of new collection and sequencing efforts.

**FIGURE 1:**
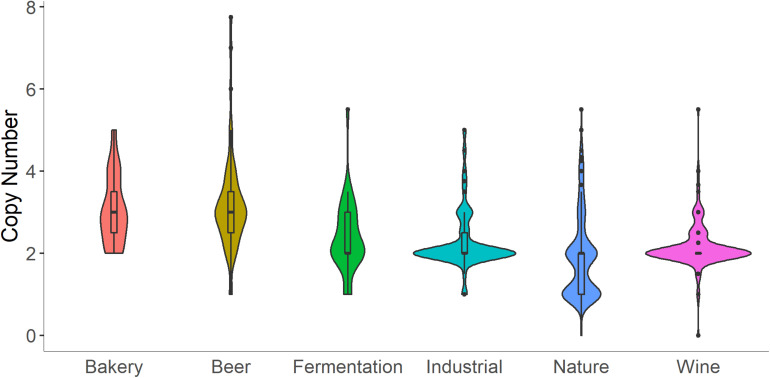
Copy number of the maltase gene, *MAL12*, is increased in bakery strains. Copy number and ecological origin data were collected from Peter et al. (2018). Here, “Fermentation” refers to fermentation separate from beer, wine, and bread, and includes isolates from processes like cacao fermentation. “Industrial” refers to processes separate from fermentation, and includes isolates from origins like bioethanol production. “Nature” refers to isolates from trees, fruit, flowers, soil, etc.

In the *S. cerevisiae* pangenome, the presence of MAL loci is variable across strains, with particular MAL genes found in most of the 1011 genomes surveyed, and some MAL genes only present in about half (Peter et al., 2018). Increased copy number of specific MAL genes appears to be a common adaptation to beer and bread environments (Figure 1; Duval et al., 2010; Bigey et al., 2020). Higher copy number of genes associated with other enzymes involved in isomaltose and sucrose utilization are also reported (Bell et al., 2001; Bigey et al., 2020). To our knowledge, no specific tests have been performed to assess the effect of increased copy number of MAL loci on dough fermentation performance. However, results from experimental evolution support the conclusion that increased copy number of nutrient transporters confers a fitness advantage in nutrient limited environments (Dunham et al., 2002; Gresham et al., 2008; Payen et al., 2014; Selmecki et al., 2015; Sunshine et al., 2015; Smukowski Heil et al., 2017). One could speculate that increases in MAL copy number could provide an advantage during fermentation, possibly increasing the speed of fermentation, or shortening the onset of fermentation, though more research is required to pinpoint the possible advantages.

In addition to the presence and copy number of MAL loci, efficient maltose utilization is heavily impacted by a regulatory system known as glucose repression. In the presence of monosaccharides, *S. cerevisiae* activates the glucose repression pathway, which shuts down aerobic respiration in favor of anaerobic fermentation (Sicard and Legras, 2011), yielding alcohol in place of CO_2_. This can be problematic in bread baking, as glucose repression will also shut down fermentation of maltose and other di- and polysaccharides. This creates a lag time, which delays the start of fermentation and decreases overall gasing ability. The duration of lag time depends on both expression and production of alternate carbon metabolizing proteins such as maltose and MAL genes, as well as how quickly yeast cells can lift glucose repression and activate respiration (Cerulus et al., 2018; Perez-Samper et al., 2018; Vermeersch et al., 2019). During this time the dough does not rise, pausing the bread baking process until the glucose is depleted and the yeast are able to switch to maltose as their main substrate. This can translate to a smaller finished loaf than a dough made using a strain with combined glucose and maltose fermentation (Olsson and Nielsen, 2000).

Glucose repression in bread dough functions through the suppression of MAL loci by the regulatory protein Mig1 (Keleher et al., 1992; Treitel and Carlson, 1995). Mig1 recognizes and binds the MAL promoters, recruiting Ssn6 and Tup1 to inhibit gene expression (Keleher et al., 1992; Treitel and Carlson, 1995). Mig1 is therefore a potential target to increase the speed and efficiency of maltose utilization in the presence of glucose, and thus the fermentation process as a whole. However, gene knockouts have shown that deletion of both *MIG1* and *MIG2* does not increase maltose use in bread, and in some strains, *MIG1* deletion actually increases glucose repression, indicating there are other regulating factors involved (Olsson and Nielsen, 2000). For example, deletion of *MIG1* in an industrial strain increased the effects of glucose repression on maltose, but did not enhance repression in the laboratory strain (Olsson and Nielsen, 2000). Furthermore, the *MIG1* deletion negatively affected the industrial strain’s growth rate, but did not hinder growth in the laboratory strain (Olsson and Nielsen, 2000). The non-uniform deletion response, especially between laboratory and industrial strains, indicates that there are important genetic background effects. When MAL loci are placed under constitutive promoters independent of Mig1, maltose and glucose metabolism co-occur, lowering fermentation time and increasing gassing power (Olsson and Nielsen, 2000).

With maltose utilization being a vital part of bread fermentation, continued research into methods that enhance maltose utilization are necessary. Areas that require attention include functional differences between MAL loci, the effect of copy number variation on fermentation dynamics, and strain optimization to allow co-metabolism of glucose and maltose. Better understanding of these characteristics could lead to shorter fermentation times, and increased bread volume for industrial producers.

### Managing Osmotic Stress via Glycerol Accumulation and the HOG Pathway

*Saccharomyces cerevisiae* experiences extreme osmotic shock during dough fermentation and commercial yeast processing (Aslankoohi et al., 2013, 2015; Randez-Gil et al., 2013). The semi-solid state of dough and high salt and/or high sugar recipes create hyperosmotic and ionic stress (Hernández-López et al., 2003; Aslankoohi et al., 2013; Randez-Gil et al., 2013). In the face of hyperosmotic conditions, cells lose water due to the osmotic gradient formed between both sides of the cell membrane, and growth is halted (Hohmann et al., 2007). This can result in decreased viability and decreased fermentation capacity, which manifests in longer proofing times and smaller loaves. Thus, managing osmotic stress is a fundamental property of strains used in bread baking.

*Saccharomyces cerevisiae* responds to osmotic stress through strong upregulation of *AQR1*, a membrane transporter for amino acid excretion during restrictive growth conditions (Aslankoohi et al., 2013), and through the production and accumulation of glycerol. Glycerol prevents water loss by balancing intracellular osmolarity so it more resembles the environment (Sasano et al., 2012b; Aslankoohi et al., 2013, 2015). Glycerol homeostasis is managed through the high-osmolarity glycerol (HOG) pathway, a mitogen-activated protein kinase (MAPK) pathway central in stress-activated response and signaling (Hohmann, 2009; Brewster and Gustin, 2014). The HOG pathway is well conserved, with the central player *HOG1* homologous to mammalian MAPK p38, which is involved in inflammatory and stress responses (Han et al., 1994; Raingeaud et al., 1995). Individual genes within the HOG pathway exhibit different evolutionary rates between or within lineages of fungi, with osmosensory genes upstream of *HOG1* evolving more rapidly (Nikolaou et al., 2009; Wu et al., 2010; Li et al., 2013). For example, the osmosensing transmembrane receptors *MBS2* and *SLN1* have high nucleotide diversity, and a branch-site model test to detect selection acting on two Chinese rice wine strain branches is suggestive of adaptation to osmotic stress caused by high sugar in rice wine (Li et al., 2013). In contrast, there is some evidence that osmotic adaptation to high sugar dough is not a defining feature of bakery strains, and instead, osmotolerance is variable across both commercial baking strains and non-bakery strains (Bell et al., 2001). Whether the bakery strains that can ferment in high sugar dough do so as a result of selection on osmo-receptors has not yet been examined.

The HOG pathway response to osmotic shock is transitory, with the cell strongly suppressing *HOG1* once the cell stabilizes (Hernández-López et al., 2003; Hohmann et al., 2007). How fast strains can respond to osmotic stress, and whether the response is maintained or not, may impact dough fermentation dynamics. One study found that baking strains of another yeast, *Torulaspora delbrueckii*, responded faster to osmotic stress, with a faster increase in glycerol levels, out performing two commercial *S. cerevisiae* baking strains in high sugar doughs (Hernández-López et al., 2003). The *T. delbrueckii* strains also decreased glycerol concentrations after the initial inoculation, whereas the *S. cerevisiae* strains maintained high glycerol concentrations throughout fermentation. This may represent another trait that could be further optimized in *S. cerevisiae* baking strains.

Genes involved in glycerol homeostasis are some of the most differentially upregulated genes during the onset of dough fermentation and are essential for yeast growth in dough (Aslankoohi et al., 2013). When cells encounter osmotic stress, *HOG1* induces expression of the glycerol pathway genes *GPD1*, *GPP1*, and *GPP2*. *GPD1*, the first enzyme in the synthesis pathway of glycerol, is key in glycerol content and successful dough fermentation (Albertyn et al., 1994). Strains and species with different glycerol production levels often show differences in expression of *GPD1* and/or Gpd1 enzymatic activity (Attfield and Kletsas, 2000; Oliveira et al., 2014), although this has not been systematically surveyed in diverse baking strains. *GPD1* has thus been a target for genetic manipulation to modulate glycerol accumulation. Deletion of *GPD1* results in decreased glycerol concentration, reduced CO_2_ production, and delays in dough fermentation (Aslankoohi et al., 2013, 2015). Overexpression of *GPD1*, on the other hand, can increase fermentation rates in high-sugar dough and improve dough gas retention, although improvements are more stark for laboratory strain backgrounds than bakery strain backgrounds (Barrett et al., 2000; Baik and Chinachoti, 2002; Styger et al., 2011; Aslankoohi et al., 2015; Heitmann et al., 2018). This is suggestive that some baking strains are indeed better adapted to dough conditions, and produce dough with better gas retention, due to higher base levels of glycerol amongst other selected traits.

The glycerol proton symporter *STL1* is also significantly upregulated upon the start of dough fermentation. *STL1* is part of a glycerol uptake system that imports glycerol from the environment to increase internal glycerol concentrations (Ferreira et al., 2005; Hohmann et al., 2007; Duskova et al., 2015). Stl1 functions together with the glycerol export protein Fps1 to control intracellular glycerol content and modulate glycerol leakage into the dough (Oliveira et al., 2003; Thorsen et al., 2006; Hohmann et al., 2007; Hohmann, 2009; Randez-Gil et al., 2013). Some glycerol leakage is beneficial, as it softens and relaxes the dough, increasing its ability to contain CO_2_ and thus increasing overall dough rise (Aslankoohi et al., 2015). However, excessive glycerol in dough can have a negative effect on bread aroma and taste (Olsson and Nielsen, 2000). Glycerol levels also affect the shelf-life of finished loaves (Barrett et al., 2000; Baik and Chinachoti, 2002; Styger et al., 2011; Heitmann et al., 2018).

### Trehalose and Proline Accumulation Protect Against Osmotic, Freeze, and Desiccation Stress

In addition to osmotic stress, baking yeast are subject to a variety of other stressors, particularly related to industrial manufacturing and distribution processes. For example, frozen dough is used to provide easier access to fresh-baked bread for consumers while balancing labor conditions for bakers and allowing for greater geographic distribution of products (Hsu et al., 1979; Luo et al., 2018). Typical baking strains fail to retain leavening ability following freezing, and thus cryotolerant strains have been isolated from natural environments, or developed through genetic modifications in the lab (Hino et al., 1987; Hahn and Kawai, 1990; Matsutani et al., 1990; Nakagawa and Ouchi, 1994; Takagi et al., 1997; Shima et al., 1999). Much of the attention in freeze tolerant baking strains has focused on the naturally occurring cryoprotectants trehalose and proline, which protect cells from a variety of stresses including osmotic stress, freezing, dehydration, and heat shock (Shima et al., 1999; Sasano et al., 2012a). Trehalose and proline accumulation allow commercial baking yeast to survive processing and distribution either in dehydrated, dry yeast or frozen in pre-made dough (Sasano et al., 2010, 2012b; Randez-Gil et al., 2013). We address current knowledge of trehalose and proline accumulation in dough in turn, below.

Trehalose is a sugar composed of two glucose molecules linked at their 1-carbons. The cellular concentration of trehalose is balanced by the relative rates of its synthesis and degradation. Trehalose-6-phosphate synthetase (*TPS1*) and trehalose-6-phosphate phosphatase (*TPS2*) synthesize trehalose in the cytoplasm (Bell et al., 1992, 1998), while neutral trehalase (*NTH1*) and acid trehalase (*ATH1*) breakdown trehalose (Kopp et al., 1993; Alizadeh and Klionsky, 1996). High levels of trehalose are strongly correlated with high levels of stress tolerance (Attfield, 1997), however, the trehalose content in commercial baking strains varies considerably (Lewis et al., 1997). Deletion of one or both *NTH1* and *ATH1* increases trehalose concentrations and gassing power of frozen doughs, with the *NTH1* deletion providing the most freeze protection. This has made *NTH1* a common target for creation of freeze-tolerant baking strains (Shima et al., 1999; Dong et al., 2016; Xi et al., 2016; Takagi, 2017). Deletion of one, but not both *NTH1* and *ATH1* also increased the yeast’s tolerance of dry conditions (Kim et al., 1996; Shima et al., 1999).

The amino acid proline functions in stress response across many organisms (Csonka and Hanson, 1991; Delauney and Verma, 1993). Proline stabilizes proteins and membranes, lowers the T_*m*_ of DNA, and scavenges reactive oxygen species (ROS), which is believed to be a main killer of yeast in osmotic, drying, and freezing stress (Samuel et al., 2000; Takagi, 2008; Sasano et al., 2010, 2012b). However, proline is not naturally elevated in response to these stressors in *S. cerevisiae*, and instead appears to be constitutively expressed (Brandriss and Falvey, 1992; Samuel et al., 2000; Kaino and Takagi, 2008; Takagi, 2008; Sasano et al., 2010, 2012a,b; Randez-Gil et al., 2013). Nevertheless, researchers have demonstrated that synthetically increasing *S. cerevisiae* intracellular proline levels by making genetic modifications to the proline synthesis and breakdown pathways confers higher freezing and desiccation tolerance, and better fermentation performance in frozen and sweet doughs (Nomura and Takagi, 2004; Sekine et al., 2007; Sasano et al., 2012a, b; Randez-Gil et al., 2013; Steensels et al., 2014b; Tsolmonbaatar et al., 2016). Other efforts have shown that different alleles of the genes *MPR1* and *MPR2*, which detoxify the toxic proline analog azetidine-2-carboxylate, also are involved in proline accumulation and mitigating desiccation stress and cryotolerance (Nomura and Takagi, 2004; Sasano et al., 2010).

Intriguingly, efforts to simultaneously increase levels of both trehalose and proline have yielded higher tolerance to oxidative and freezing stresses and improved the fermentation ability in dough after being frozen compared with the singular accumulation of proline or trehalose (Sasano et al., 2012a). This work suggests that proline and trehalose protect yeast cells from short-term and long-term freezing effects, respectively, and is an interesting area for further pursuit which could be beneficial to the frozen dough industry.

### Aromatic Compound Production

The sensory qualities of bread, such as aroma and taste, are essential metrics of quality for consumers, and are strongly influenced by volatiles and secondary metabolites produced by yeast (Schieberle and Grosch, 1991; Frasse et al., 1993; Olsson and Nielsen, 2000; Birch et al., 2013a, b; Pico et al., 2015; Aslankoohi et al., 2016; Dzialo et al., 2017) The identity and relative abundance of aroma compounds vary widely among strains of *S. cerevisiae*, and, more broadly, across species of yeasts (Christiaens et al., 2014; Steensels et al., 2014b). Variation in aromas may relate to the adaptive diversification of yeast strains and species in as much aroma compounds play important physiological and ecological roles in yeasts, including regulation of growth, communication, and signaling to insect vectors (Richard et al., 1996; Bruce et al., 2004; Leroy et al., 2011; Becher et al., 2012; Davis et al., 2013). The attraction of insect vectors has been shown to mediate important yeast life history traits including outcrossing and dispersal (Reuter et al., 2007; Christiaens et al., 2014; Stefanini et al., 2016; Madden et al., 2018). As a result, non-human animals may be important in engendering the diversity and abundance of aromas produced among yeast strains. Recent studies support the hypothesis that domestication of *S. cerevisiae*, by humans, for various industrial applications has favored desirable aroma compounds, and disfavored off-flavors in bread and other fermented food (Wedral et al., 2010; Suárez-Lepe and Morata, 2012; Gallone et al., 2016; Padilla et al., 2016; Fay et al., 2019; Langdon et al., 2019).

Different sourdough and commercially available baking strains of *S. cerevisiae* can generate significantly different aroma profiles in bread (Birch et al., 2013a; Pétel et al., 2017). The more influential aroma compounds include alcohols, aldehydes, ketones (e.g., acetoin and diacetyl), and esters (e.g., ethyl acetate), which are in part regulated via the Ehrlich metabolic pathway (Styger et al., 2011; Birch et al., 2013a; Pétel et al., 2017). Typically, ethyl acetate has an aroma similar to pineapple, diacetyl and acetoin are buttery, alcohols and aldehydes provide floral and sometimes fruit notes, and esters, particularly saturated esters, are fruity in nature (Fingolfn Practically Science, 2013). Combined, these molecules provide the aromatic qualities of each loaf, and can be quantified and analyzed to detect variation across strains. One such study assessed aromas produced by seven different *S. cerevisiae* bakery strains and found that aroma compounds varied by an order of magnitude between strains for compounds like 3-methylbutanal, which has a malty aroma, and 2,3-butanedione, which has a buttery aroma. While not all compounds had such ranges, the variety of aroma concentration would provide each bread with a unique aroma profile, or lack thereof (Aslankoohi et al., 2016; Reese et al., 2020). This is compounded by the fact that different compounds have different odor detection thresholds (ODT), or the concentration at which the human nose can detect it in water. The range of aroma compounds and their concentration could overlap with the compounds’ ODT. Indeed, one strain was found to have significantly less of almost all aroma compounds tested, meaning the bread would have less distinct scent compared to bread prepared with one of the other commercial baking yeasts, as it would only have aromas from the flour and maillard reaction from the baking process.

Improving flavor and aroma in baked products is an active area of research, and includes a variety of techniques including experimental evolution, gene modifications and exploiting natural diversity (Dzialo et al., 2017), which we address in more depth below.

## Future Needs

Current commercial baking strains are not optimized for all desired baking and processing traits, as industry often uses strains due to historical reasons (Steensels et al., 2014a). Some common categories where additional optimization is needed include: increased fermentation capacity in sweet doughs, resistance to salt toxicity, better storage survival (frozen and dried), enhanced sensory qualities such as taste, aroma, and texture, synthesis of beneficial and functional metabolites such as antioxidants, phenols, xanthophyll, and anthocyanins, and microbial stability (Becker et al., 2003; Hernández-López et al., 2003; Wang et al., 2011; Steensels et al., 2014a; Palla et al., 2020). A strain that is capable of fermenting both standard lean dough and sweet doughs is also highly desirable (Hernández-López et al., 2003). Past and current efforts to meet these demands are summarized here through efforts to exploit natural diversity (bioprospecting) and genetic modification of existing strains (bioengineering).

### Bioprospecting

Bioprospecting generally describes the search for new strains or species with beneficial characteristics that could be leveraged in industry. Bioprospecting of *S. cerevisiae* holds considerable promise considering the species’ genetic and phenotypic diversity, with documented variation in many baking traits, including maltose utilization, aroma compounds, trehalose content, glycerol content, and general stress tolerance (Steensels et al., 2014b). Potential sources for baking strain bioprospecting can be generally divided into wild and man-made environments. Bioprospecting has already been employed to identify freeze-tolerant strains for use in baking (Hahn and Kawai, 1990), and there are numerous known cryotolerant species of *Saccharomyces* (Salvadó et al., 2011; Sylvester et al., 2015), some of which have already been utilized in cold fermented wines, beers, and ciders. Insects represent another promising potential source for yeast bioprospecting in natural environments, with insect-isolated yeasts already proving to be viable options in bioethanol production, beer, and other industrial uses (Urbina et al., 2013; Steensels and Verstrepen, 2014; Sheppard et al., 2015; Mohd Azhar et al., 2017; Madden et al., 2018). In addition to isolating environmental yeasts from living in similar habitats to industrial conditions, there is the potential to use “contaminating” strains (e.g., contaminants in wine and beer), or spontaneous inoculated strains (e.g., beer, bread, etc.) which have evolved to survive in the desired environment and have potentially beneficial phenotypes for industry (Steensels et al., 2014a). This could be especially relevant for the baking industry, as wild-yeast fermented breads like sourdough, injera, Indian flatbreads, and Chinese steamed bread are teeming with microbial diversity (Zhang et al., 2011; Li et al., 2016; Tamang et al., 2016; Liu et al., 2018; Tadesse et al., 2019; Koricha et al., 2020). The *S. cerevisiae* strain diversity in these homemade grain ferments is largely unknown, and what is known is predominantly from European and North American isolates. Sampling from worldwide wild-yeast fermented breads, with a focus to increase representation from Africa, Asia, and South America, should be a priority for finding desirable baking strains in the future.

### Bioengineering

Bioprospecting and bioengineering should not be considered mutually exclusive, but generally, bioengineering takes a more direct method in creating strains with beneficial phenotypes. Here, we will also use this term to encompass traditional breeding techniques. Somewhat surprisingly, selective breeding has not been heavily employed for industrial yeast strain improvement, despite the genetic and phenotypic variation present (see reviews Steensels et al., 2014a; Cubillos, 2016). Certainly there are challenges to traditional crosses, industrial *S. cerevisiae* strains are more prone to polyploidy and aneuploidy, and thus have a much lower spore viability than lab strains. Large scale screening for desirable traits in bread baking also presents practical challenges, like the ability to phenotype many individual crosses for fermentative traits and aroma compounds.

Despite these obstacles, the genetic mapping of complex traits in *S. cerevisiae* utilizing wild, clinical, lab, and industrial strains has been hugely successful (Ehrenreich et al., 2009; Liti and Louis, 2012; Swinnen et al., 2012; Gutiérrez et al., 2013; Jara et al., 2014; Wilkening et al., 2014; Bloom et al., 2019; Fournier et al., 2019), and underscores the ability to utilize direct crosses with the potential for selective breeding. There have been a few efforts to cross in useful traits for production of beer (Nikulin et al., 2018) and biofuels (Benjaphokee et al., 2012), and the wine industry in particular has used crossing, with a particular emphasis on interspecific hybridization with other *Saccharomyces* species. Hybrid crosses have been utilized in the wine industry to impart new flavors/aromas (Bellon et al., 2011, 2013; Kanter et al., 2019) and freeze-tolerance (Kishimoto, 1994; Zambonelli et al., 1997; Sipiczki, 2008; Pérez-Través et al., 2012), although these hybrids are typically sterile. New genetic editing techniques that allow for the successful completion of meiosis in normally sterile hybrids (Bozdag et al., 2019) is a promising development for the future use of interspecific hybrids in industry.

Finally, genetic modifications through gene deletions, allele replacements, and the insertion of new genetic materials have been successfully used to create baking strains with better fermentation dynamics and stress tolerance. We have highlighted many studies in this review that have utilized genetic modifications to better understand how individual genes or pathways contribute to desirable and undesirable traits in bread baking. However, moving these modified strains from research labs to the bakery presents major hurdles. Industrial use of genetically engineered organisms in food is illegal or highly regulated in most countries (Steensels et al., 2014a), and requires a change in consumer opinion of genetically modified organisms (GMO). In this regard, other common techniques like mutagenesis and directed laboratory evolution may hold more applicable potential. These methods have been applied to wine (Bellon et al., 2018; Mangado et al., 2018), biofuel (Sato et al., 2016; Peris et al., 2017), and beer (Baker and Hittinger, 2019), and to increase stress tolerance (Tsolmonbaatar et al., 2016) and freeze-tolerance in baking strains (Aguilera et al., 2010). A future that exploits natural variation through bioprospecting, traditional crosses, and directed laboratory evolution may help meet both consumer and baker preferences.

## Conclusion and Outstanding Questions

There are clear desires of bakers and consumers for more flavorful, nutritious breads, and bakers need strains that show increased osmotolerance, cryotolerance, and desiccation resistance without the loss of fermentation capacity. In this review, we have outlined the genetic and phenotypic diversity of *S. cerevisiae* baking strains. We note that many researchers have documented variation in traits important for baking, including maltose utilization, trehalose content, glycerol content, aroma compounds, freeze tolerance, osmotolerance, and fermentation metrics like total CO_2_. Most of these studies have only used a small handful of strains, which suggests we have only surveyed a portion of the phenotypic variation that may exist. With many more isolates being collected from home, artisanal, and commercial bakers, there is an opportunity to better understand the evolutionary history of baking strain domestication and molecular evolution and selection on gene variants; map genetic loci contributing to complex traits; and develop better baking strains. We conclude with the following outstanding questions that can serve as a guide for future research.

Outstanding Questions:

Why are interspecies hybrids repeatedly found in beer and wine, but not bread?
What is the genetic diversity and biogeography of *S. cerevisiae* strains used in sourdough starters in the home?
Are there molecular signatures of selection in baking strains?
Do signatures of domestication differ between fermented breads from different cultures?

## Author Contributions

CL and CSH were responsible for the writing and editing of this manuscript. RD and AM were responsible for the editing of this manuscript. All authors contributed to the article and approved the submitted version.

## Conflict of Interest

The authors declare that the research was conducted in the absence of any commercial or financial relationships that could be construed as a potential conflict of interest.
